# Supplementary material for: The Heterogeneous Habitat of Taiga Forests Changes the Soil Microbial Functional Diversity
Source: Microorganisms. 2024 May 10;12(5):959. doi: 10.3390/microorganisms12050959 (PMC11124070; doi:10.3390/microorganisms12050959)
Supplement: Supplementary file 1 [file microorganisms-12-00959-s001.zip › microorganisms-2983797-supplementary.pdf]

Supplementary materials:

Table S1. Grouping situation of taiga forests. All data are displayed as mean ± standard error (n = 3), and different letters in the same column denotes significant difference in taiga forests with different important values ($p < 0.05$).

Experimental group	Important value
L1	0.434±0.009 ^{e}
L2	0.481±0.007 ^{d}
L3	0.514±0.012 ^{c}
L4	0.544±0.007 ^{b}
L5	0.585±0.009 ^{a}

Table S2. Differences in soil physical and chemical properties in taiga forests. All data are displayed as mean ± standard error (n = 3), and different letters in the same column denotes significant difference in taiga forests with different important values ($p < 0.05$).

Experimental Group	TN (g/kg)	AK (mg/kg)	AP (mg/kg)	AN (mg/kg)	SOC (g/kg)	MBC (mg/kg)	pH	WC (%)	C/N
L1	2.14 ± 0.02 ^{b}	137.44 ± 0.92 ^{c}	20.23 ± 0.08 ^{e}	67.57 ± 0.13 ^{c}	73.66 ± 1.46 ^{a}	200.77 ± 0.09 ^{a}	5.48 ± 0.01 ^{a}	31.16 ± 1.07 ^{b}	34.39 ± 0.73 ^{a}
L2	2.15 ± 0.02 ^{b}	112.21 ± 0.68 ^{e}	45.25 ± 2.02 ^{d}	69.47 ± 0.17 ^{c}	55.54 ± 0.33 ^{b}	170.70 ± 0.06 ^{b}	5.14 ± 0.01 ^{b}	34.77 ± 0.53 ^{ab}	25.80 ± 0.29 ^{b}
L3	2.27 ± 0.01 ^{a}	191.49 ± 0.97 ^{a}	57.16 ± 0.65 ^{c}	79.68 ± 1.46 ^{b}	42.18 ± 0.53 ^{c}	147.77 ± 0.03 ^{c}	5.05 ± 0.00 ^{c}	36.35 ± 2.35 ^{a}	18.58 ± 0.27 ^{c}
L4	2.29 ± 0.00 ^{a}	188.15 ± 0.62 ^{b}	61.56 ± 1.34 ^{b}	96.13 ± 1.17 ^{a}	40.29 ± 0.11 ^{cd}	139.67 ± 0.15 ^{d}	4.41 ± 0.01 ^{d}	36.06 ± 1.18 ^{a}	17.59 ± 0.05 ^{cd}
L5	2.31 ± 0.01 ^{a}	131.45 ± 0.70 ^{d}	77.38 ± 0.75 ^{a}	98.35 ± 0.70 ^{a}	39.41 ± 0.22 ^{d}	116.40 ± 0.20 ^{e}	4.31 ± 0.00 ^{e}	36.86 ± 1.08 ^{a}	17.09 ± 0.07 ^{d}
